# Dual-generative synthesis framework: enhancing polyp segmentation in colonoscopy via mask-conditional GANs

**DOI:** 10.3389/fonc.2026.1887904

**Published:** 2026-07-20

**Authors:** Mejdl Safran, Sultanul Arifeen Hamim, M. F. Mridha, Dunren Che, Sultan Alfarhood

**Affiliations:** 1 Department of Computer Science, College of Computer and Information Sciences, King Saud University, Riyadh, Saudi Arabia; 2 Department of Computer Science, American International University Bangladesh, Dhaka, Bangladesh; 3 Department of Electrical Engineering and Computer Science, Texas A&M University-Kingsville, Kingsville, TX, United States

**Keywords:** conditional GAN, dual-generative synthesis, mask-conditional GAN, medical image segmentation, polyp segmentation

## Abstract

**Background and Objective:**

Colorectal cancer screening through colonoscopy relies on the segmentation of polyps, which becomes difficult when polyps are small in size and flat in shape, with subtle edges, poor contrast, and large variability in appearance. Although new deep learning techniques have improved segmentation performance, most techniques have focused on architectural innovations, leaving aside the issue of limited diversity in the training set. Although generative methods have improved, they often suffer from the lack of control over structure, misalignment of masks and images, and increased computation, making them inefficient in the case of small polyps, where they are most relevant. This study aims to present a dual generative synthesis framework for data-centric augmentation to improve segmentation performance.

**Methods:**

The proposed framework divides the problem of structure and appearance into two steps. First, procedural generation is used to create realistic masks of small, flat polyps. Second, a mask-conditioned GAN generates colonoscopy images that match the generated masks in texture and lighting conditions. This process produces anatomically realistic and perfectly aligned mask–image pairs. The generated data are incorporated into a U-Net-based segmentation model and evaluated on public polyp segmentation datasets with extensive ablation studies.

**Results:**

The proposed method achieved a Dice score of 0.8786 and an Intersection over Union (IoU) of 0.7835. The model also obtained a precision of 0.8930 and a recall of 0.8648, which are significantly higher than those of the baseline U-Net model.

**Conclusions:**

The dual generative synthesis framework improves segmentation robustness by generating realistic and aligned training data for small and flat polyps. The results demonstrate its potential to enhance the reliability of automated polyp segmentation in colonoscopy images.

## 1 Introduction

Colorectal cancer is one of the most significant causes of cancer-related mortality, and colonoscopy is an essential procedure in reducing the occurrence of this disease as well as preventing the progression of cancer in the colon and rectum. In the course of colonoscopy, the removal of polyps reduces the risk of cancer significantly Dorjsembe et al. (1)Macháček et al. (2)Yoon et al. (3). In the past few years, segmentation models based on deep learning have achieved remarkable performance in assisting doctors in automatically segmenting polyp areas in endoscopic images. U-Net-based segmentation models have achieved remarkable accuracy in the segmentation of well-defined polyps of considerable sizes Zhang et al. (4)Jha et al. (5). However, these models have faced considerable challenges in the segmentation of small polyps, which often have low contrast, flat, and poorly defined boundaries. The most significant cause of the failure of segmentation models is the unavailability of sufficient training data, as publicly available colonoscopy datasets, including popular benchmarks, have a large proportion of well-defined, protruding polyps, while small polyps are fewer in comparison Mazumdar et al. (6). Therefore, segmentation models trained on such datasets often suffer from bias in favor of large polyps, which may not perform well on small polyps, which are often significant in the context of colonoscopy. Data augmentation techniques, such as horizontal/vertical flipping, rotation, and changing the brightness of the image, do not create new anatomical structures, making it difficult to achieve significant improvement in the detection of polyps of varying sizes Liu et al. (7)Song and Shin (8).

To overcome boundaries mapping constraints, recent state-of-the-art developments have heavily prioritized complex structural and multi-task fusion mechanisms. For instance, Abidin et al. (9) developed a dual-task hierarchical feature refinement and fusion network optimized for both surgical instrument and lesion tracking, demonstrating the efficacy of cross-layer feature distillation. Similarly, Jafar et al. (10) introduced CR-Net, a high-performance semantic framework utilizing VGG16 backbones coupled with horizontal dense connections to retrieve overlooked fine-grained features during downsampling transitions. While these complex topological innovations yield precise segmentation tracking, they focus entirely on model infrastructure. They leave the downstream core vulnerable to the underlying distribution gaps present in highly imbalanced real training cohorts, emphasizing the necessity of a data-centric intervention. The potential for generative models to provide an optimistic solution for the lack of data in the field of medical images can be seen. Although the potential for diffusion models and GANs can provide realistic images, these images often come at the cost of high computation or unstable training. More importantly, many generative models lack control over lesion structure, which is critical for segmentation since precise spatial alignment between images and masks is needed Bernal et al. (11). Therefore, this work presents a Dual-Generative Synthesis (DGS) framework that aims to improve segmentation for small, flat polyps. Rather than relying on a single model, this approach divides the process into two stages. First, it generates anatomically plausible lesion masks that include size, shape, and irregular borders of small polyps. Second, it uses a GAN to produce realistic images of the colonoscopy, ensuring that the generated lesions have precise spatial alignment between images and masks Lin et al. (12).

Unlike existing generative paradigms in medical imaging, the proposed framework introduces three critical distinctions that highlight its novelty. First, in contrast to standard GAN-based augmentations that synthesize tissue structures and surface anomalies simultaneously—often resulting in spatial drift or mode collapse for small lesions—our architecture explicitly separates geometry from texture through a decoupled pipeline. Second, compared to diffusion-based architectures (such as DDPMs or Latent Diffusion Models) that require intensive, multi-step iterative sampling, our framework yields comparable textural authenticity while maintaining an efficient, single-pass inference mechanism optimized for high-throughput data expansion. Third, while traditional mask-conditioned models rely on simple geometric transformations or manual tracings of existing annotations, our approach leverages a procedurally driven mask generation process that mathematically simulates natural mucosal border irregularities, creating entirely unique and anatomically variable training samples. While basic convolutional encoders and standard adversarial architectures represent established structural tools in computer vision, their implementation inside our framework provides a specific operational benefit. Rather than aiming for high internal architectural complexity—which dramatically increases latency overhead—the novelty of this work lies in its decoupled, data-centric generative pipeline. By using a non-deep learning procedural engine to establish immutable spatial constraints prior to deep texturing, we resolve the severe spatial drift and mode collapse that routinely undermine advanced diffusion networks when synthesizing tiny target masks, providing a highly scalable and streamlined performance edge. The generated synthetic data are integrated with real and traditionally augmented images to train a segmentation network based on a U-Net architecture with attention mechanisms and multi-scale feature aggregation. Through this data-centric enhancement, the model becomes more sensitive to subtle lesion patterns without sacrificing overall segmentation accuracy or training stability.

The main contributions of this work are summarized as follows:

- A dual-generative synthesis framework that separately models lesion geometry and visual appearance to create realistic small-polyp training samples.
- A mask-conditioned GAN with segmentation consistency guidance, which ensures that produced images are anatomically aligned with their respective masks.
- A data-driven strategy to rebalance lesion representation, improving sensitivity to small and sessile polyps without collecting additional clinical data.
- Comprehensive validation, including quantitative evaluation, ablation studies, qualitative analysis, and explainability assessment, demonstrating improved performance and model interpretability.

The remainder of this paper is structured as follows. Section 3 reviews related work. Section 4 describes the proposed framework and Section 5 details the evaluation protocol and validation design. Section 6 presents experimental results, while Section 7 discusses key findings. Finally, Section 8 concludes the paper with future directions.

## 2 Related work

Early deep learning approaches for polyp segmentation focused on improving feature representation through architectural refinements of encoder–decoder networks. PraNet introduced reverse attention for progressive refinement and achieved Dice scores of 0.898 on Kvasir-SEG and 0.832 on ColonDB Lin et al. (12). However, its performance degraded on more challenging datasets containing small or low-contrast lesions, revealing sensitivity to boundary ambiguity and domain shift. Attention-enhanced architectures further improved localization by emphasizing salient regions. XPolypNet integrated explainable AI into a U-Net framework and reported improved interpretability alongside competitive segmentation accuracy, though gains were mainly observed on datasets dominated by large polyps Arnob et al. (13). Attention-based multi-scale models such as Attention-ASPP U-Net variants achieved Dice scores above 0.83 and precision near 97% on selected datasets Song and Shin (8). Despite these improvements, CNN-attention models remain limited in detecting subtle or sessile lesions due to insufficient representation of such cases during training.

Considering the fact that the delineation of contours has a significant impact on the reliability of clinical applications, researchers are increasingly preferring boundary-aware and uncertainty-aware models. Dual boundary-guided attention was proposed by DBE-Net Ma et al. (14) for improved performance, while multi-level attention was utilized for boundary guidance by BMANet Wu et al. (15). More recent developments, such as BGGL-Net Liu et al. (16), report a 12.4% improvement over previous models for the detection of small polyps, although the improvement is achieved at the cost of complex pipelines. Even though these models, along with two-stage models like DUMNet Zhang et al. (4), considerably improve performance in complex regions, they often come at the cost of real-time capability due to the complex nature of their pipelines.

To balance accuracy with clinical deployment needs, research has emphasized computational efficiency. Models like GFANet Lin et al. (12) and DDCNet Tong et al. (17) utilize hierarchical fusion and dual-domain modeling to maintain accuracy while improving efficiency. In the realm of lightweight architectures, LGPS Tesema et al. (18) introduced a GAN-based framework featuring a MobileNetV2 backbone and Convolutional Conditional Random Fields (ConvCRF) for boundary refinement. With only 1.07 million parameters, LGPS Tesema et al. (18) achieves a Dice score of 0.7299 on the challenging PolypGen dataset, underscoring the potential for real-time diagnostic support on resource-constrained hardware.

Data imbalance has been addressed in the literature by exploring the use of generative augmentation. For instance, early attempts in GAN-based colonoscopic image synthesis have shown improved detection of sessile serrated lesions by including the synthesized images in the training process, yielding significant improvements in sensitivity Yoon et al. (3). Semantic image synthesis of polyps and the use of GAN- based image synthesis have shown improvements in the performance of the system, including improvements in F1/Dice of over 4% in various detection and segmentation settings Song and Shin (8)Arnob et al. (13). More recently, diffusion-based image synthesis has emerged as an alternative. Polyp-DDPM has shown the efficacy of the approach by generating diverse synthetic images of polyps for segmentation, yielding 83.42% in the augmentation setting Dorjsembe et al. (1). Similarly, mask-conditioned latent diffusion has shown improvements in the realism of the images, yielding 84.65% in the augmentation setting Macháček et al. (2). However, the approach is computationally expensive, making it slow. The use of lightweight GANs, such as LGPS, has shown the efficacy of the approach by yielding 72.99% in the segmentation setting, requiring as few as 1.07M parameters Liu et al. (7)Tesema et al. (18). However, the approach is not effective in the segmentation setting. Similarly, large-scale dataset expansion, such as Polyp-Gen, has shown the efficacy of the approach by yielding realistic and diverse images, although the approach did not consider the alignment of the masks.

Despite extensive research, key limitations remain in polyp segmentation. CNN and attention-based models achieve Dice scores near 0.90 on easier datasets but struggle with small and sessile polyps. Boundary-aware approaches improve contour precision by only a few percentage points while introducing complex pipelines. Uncertainty-aware models enhance robustness (e.g., Dice 0.766 on ETIS) but increase computational burden. Multi-scale and transformer-based methods strengthen contextual modeling yet remain computationally expensive. Generative augmentation shows Dice gains of 3–5%, but most methods lack structural control, fail to ensure mask–image alignment, or require heavy computation. To address these issues, this work proposes a dual-generative synthesis framework for structure-aware, mask-aligned polyp data generation. To clearly contextualize the unique positioning of our method against the current literature, **Table 1** synthesizes the core differences across major generative augmentation paradigms.

**Table 1:** Taxonomy and architectural comparison of the proposed framework against existing generative synthesis paradigms for polyp data augmentation.

Property	Standard GANs	Diffusion models	Traditional mask-cond.	Ours (DS-GPF)
decoupled structure-texture	X	X	✓	✓
Computational Efficiency	✓	**X**	✓	✓
Pixel-Level Boundary Integrity	**X**	**X**	✓	✓
Stochastic Shape Novelty	✓	✓	**X**	✓
Clinical Control for Small Lesions	**X**	**X**	**X**	✓

## 3 Methods

### 3.1 Proposed dual-generative synthesis framework

**Figure 1:**
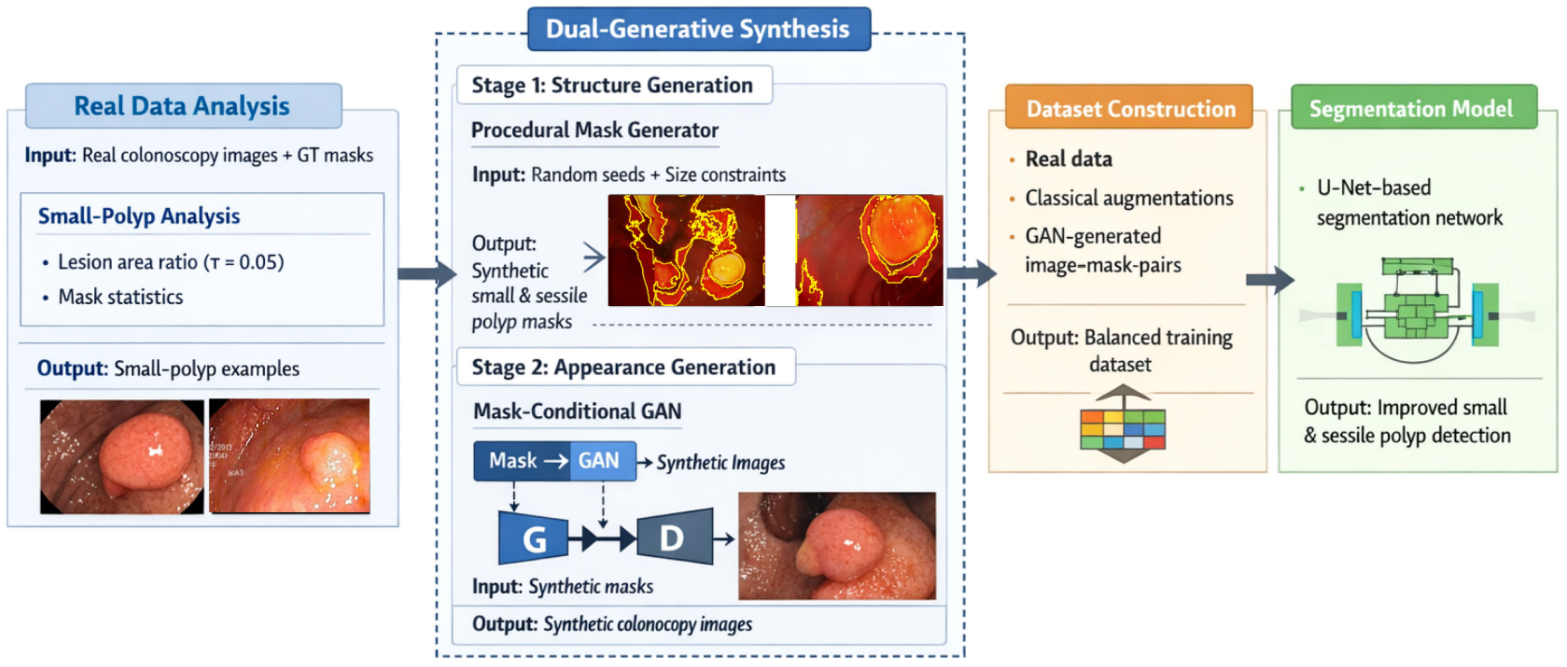
Overview of the proposed dual-generative synthesis framework for enhancing small and sessile polyp segmentation. The framework first analyzes real colonoscopy masks to identify under-represented small polyp regions based on lesion area statistics. In Stage 1, a procedural structure generator produces synthetic small-polyp masks with controlled geometry. In Stage 2, a mask-conditional GAN translates these masks into realistic colonoscopy images while preserving exact mask–image alignment. The generated synthetic samples are combined with real and classically augmented data to construct a balanced training set, which is then used to train the segmentation network, resulting in improved detection of subtle and small polyps.

To address the scarcity of small and sessile polyps in training data, this work proposes a Dual-Generative Synthesis framework that separates structural mask generation from appearance synthesis. The overall pipeline of the proposed method is illustrated in **Figure 1**. Instead of modifying the segmentation architecture itself, the proposed approach focuses on reshaping the training data distribution to better reflect clinically challenging cases.

The proposed pipeline operates via a fully decoupled, data-centric architecture structured into two sequential processing phases. Specifically, *Stage 1: Procedural Mask Generation* employs non-adversarial mathematical morphology algorithms to establish robust spatial constraints and random semantic boundary variations. Subsequently, *Stage 2: Mask-Conditional Appearance Synthesis* leverages a conditional deep generative adversarial network to paint high-fidelity mucosal features over these synthesized layouts. By isolating macro-level geometry from microscopic surface texturing, the framework successfully suppresses pixel-level boundary drift while maximizing training set structural diversity.

#### 3.1.1 Mask generation strategy

To simulate the morphology of small and sessile polyps, we propose a synthetic mask generation process designed to create anatomically plausible lesion shapes. Instead of applying transformations to existing lesion shapes as in other augmenting techniques, this technique creates new lesion shapes, allowing the model to see a wide range of subtle polyp morphologies.

Let $M\in {\left\{0,1\right\}}^{H\times W}$ denote a binary lesion mask, where a pixel value of 1 indicates the polyp region and 0 represents the background mucosa. To ensure that the generated lesions correspond to clinically small polyps, we constrain the lesion area ratio as (Equation 1)

(1) $$R_{\text{lesion}}=\frac{1}{H\times W}\sum_{i=1}^{H}\sum_{j=1}^{W}M_{ij},\;R_{\text{lesion}}<\tau ,$$

where *τ* is a predefined threshold representing the maximum allowable lesion size ratio. The rationale behind this constraint is to keep the artificial lesions within the scale range of small and sessile polyps, as observed in real colonoscopy data.

The shape of the lesions is defined as irregular blob-like objects by simulating random seed points, stochastic boundary perturbations, and smoothing. Let the boundary of the lesion mask be denoted by *∂M*. The irregularity of the boundary can be achieved by introducing spatial deformations, defined as (Equation 2)

(2) $$\partial M^{\prime }=\partial M+\epsilon \left(x,y\right),$$

where ϵ(x,y) represents a small perturbation field in space that controls the variability of the contours. This process creates non-uniform boundaries that are softly defined, as might be seen in the indistinct edges of flat or sessile lesions. Anatomical plausibility of the synthesized masks is ensured by keeping them within anatomically valid regions of the mucosa and avoiding areas close to the image edges or regions of low information content. This spatial constraint allows the generative model to learn the realistic interaction of texture with anatomical structures in the lesion. The synthesized mask acts as a structural template for the subsequent GAN-based image synthesis process. This model allows for the control of the size and shape of the lesions explicitly while learning the realistic texture, color, and illumination patterns associated with the lesions.

#### 3.1.2 GAN-based lesion appearance synthesis

**Figure 2:**
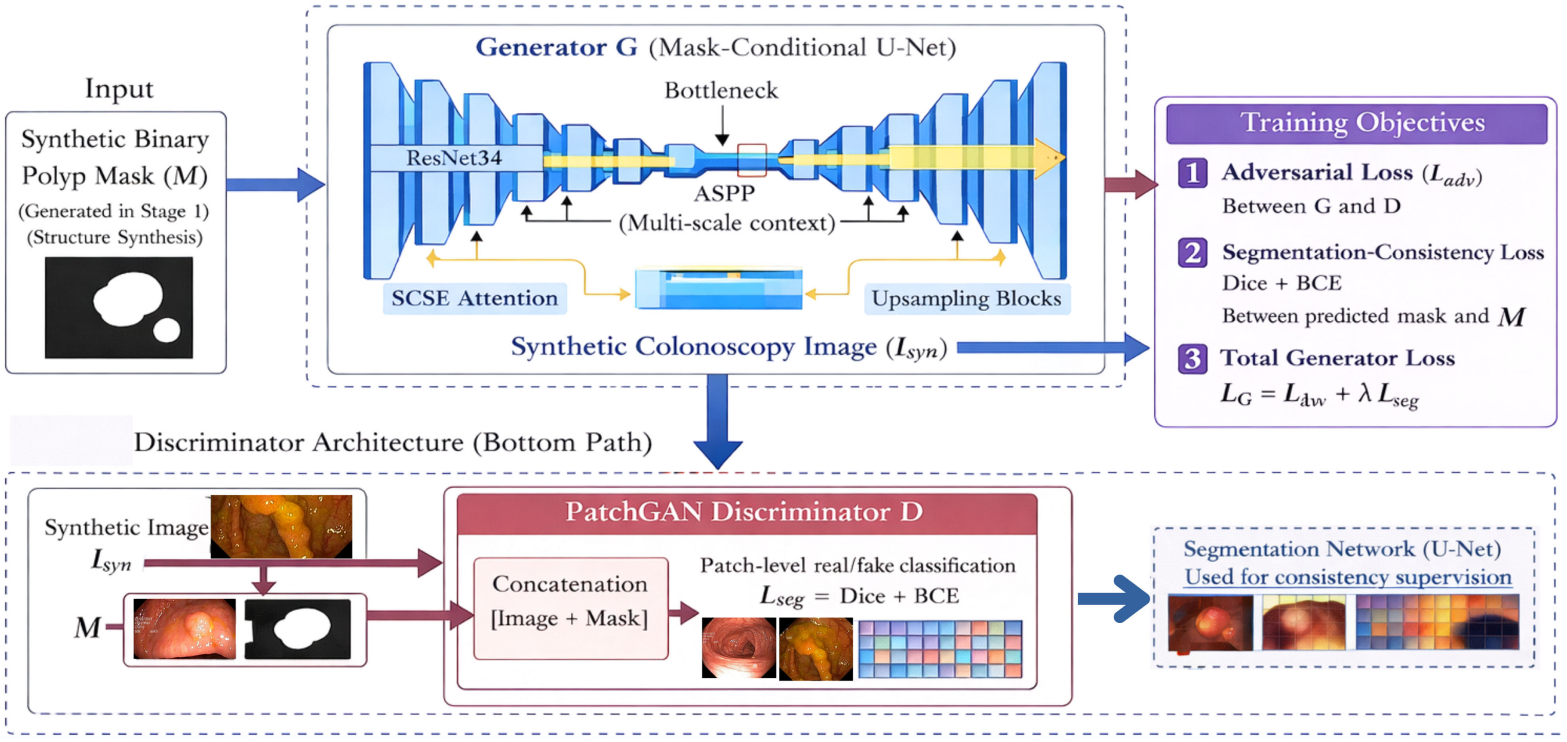
Architecture and training flow of the proposed mask-conditional GAN for lesion appearance synthesis.The generator G, implemented as a U-Net with a ResNet34 encoder, ASPP module, and SCSE attention, translates synthetic binary lesion masks M into realistic colonoscopy images I_syn_. A PatchGAN discriminator D evaluates concatenated image–mask pairs to enforce local texture realism through adversarial learning. In addition to adversarial supervision, a segmentation-consistency loss (Dice + BCE), computed using the segmentation network, guides the generator to preserve the predefined lesion geometry.

To translate the synthetically generated lesion masks into realistic colonoscopy images, we employ a mask-conditional Generative Adversarial Network (cGAN). The objective of this component is to synthesize visually plausible mucosal textures and subtle polyp appearances that are consistent with real endoscopic imagery, while remaining strictly aligned with the predefined lesion geometry. The detailed architecture of the mask-conditional GAN used for lesion appearance synthesis is illustrated in **Figure 2**.

Let *M* denote a synthetic binary lesion mask. The generator *G* learns the mapping (Equation 3)

(3) $$G:M\to I_{\text{syn}},$$

where *I*_syn_ is a synthetic colonoscopy image whose lesion appearance is spatially consistent with the input mask *M*. During training, the discriminator distinguishes between real colonoscopy images and generated samples, encouraging the synthesis of realistic mucosal textures while preserving the predefined lesion geometry.

##### 3.1.2.1 Generator architecture

The generator follows an encoder-decoder structure with skip connections, similar to a U-Net architecture. The encoder sequentially learns contextual features from the mask image, and the decoder reconstructs a high-resolution image from the encoded features. Skip connections enable the network to maintain spatial details, where lesion boundaries within the synthesized image align with the mask image. This architecture enables the network to concentrate on learning realistic texture patterns, color gradients, and illumination effects often present in colonoscopy images.

##### 3.1.2.2 Discriminator architecture

A PatchGAN discriminator *D* is employed to assess image realism at the level of local image patches rather than evaluating the image globally. Instead of producing a single scalar prediction, the discriminator outputs a grid of probabilities indicating whether each patch is real or synthetic. This strategy encourages the generator to produce high-frequency details and fine-grained textures, which are critical for modeling the subtle surface characteristics of small and sessile polyps.

##### 3.1.2.3 Adversarial learning objective

The conditional GAN is optimized utilizing a Least-Squares Generative Adversarial Network (LSGAN) objective paradigm, which leverages a quadratic loss function. This configuration minimizes gradient vanishing risks and significantly enhances high-frequency fine texture stabilization during adversarial training. The objective functions for Discriminator *D* and Generator *G* are defined respectively as (Equations 4, 5):

(4) $${ℒ}_{\text{adv}}\left(D\right)=\frac{1}{2}E_{I_{\text{real}}}\left[{\left(D\left(I_{\text{real}},M\right)-1\right)}^{2}\right]+\frac{1}{2}E_{M}\left[{\left(D\left(G\left(M\right),M\right)\right)}^{2}\right],$$

(5) $${ℒ}_{\text{adv}}\left(G\right)=\frac{1}{2}E_{M}\left[{\left(D\left(G\left(M\right),M\right)-1\right)}^{2}\right],$$

where *I*_real_ denotes a real clinical frame and *M* represents the procedurally synthesized binary semantic template.

To prevent geometric distortion and ensure structural alignment between the synthetic image space and the mask space, a bidirectional cycle consistency constraint is incorporated. An auxiliary image-to-mask parsing network *F* is introduced to map thesynthesized image back into the binary mask domain, enforcing structural reconstructibility. The Cycle Consistency Loss ( ${ℒ}_{\text{cyc}}$) is formulated as (Equation 6):

(6) $${ℒ}_{cyc}\left(G,F\right)=E_{M}\left[{∥F\left(G\left(M\right)\right)-M∥}_{1}\right].$$

Furthermore, to stabilize adversarial training dynamics and preserve fine-grained textural realism, a Discriminator Feature Matching Loss ( ${ℒ}_{\text{FM}}$) is utilized. This loss minimizes the multi-layer *L*_1_ distance between the intermediate feature map representations of real and synthetic images across *T* layers of the PatchGAN discriminator *D* (Equation 7):

(7) $${ℒ}_{FM}\left(G,D\right)=E_{\left(I_{real},M\right)}\sum_{i=1}^{T}\frac{1}{N_{i}}\left[{∥D^{\left(i\right)}\left(I_{real},M\right)-D^{\left(i\right)}\left(G\left(M\right),M\right)∥}_{1}\right],$$

where *D*^(^*^i^*^)^ denotes the internal feature extractor at the *i*-th layer of Discriminator *D*, and *N_i_* represents the total number of elements in that respective layer map.

In addition to adversarial supervision, the generator is guided by a segmentation-consistency loss that encourages the synthesized images to yield accurate polyp segmentations when processed by the segmentation network. This loss combines Dice loss and Binary Cross-Entropy (BCE) loss, and is defined as (Equation 8)

(8) $${ℒ}_{\text{seg}}\left(G\right)={ℒ}_{\text{Dice}}+{ℒ}_{\text{BCE}},$$

where ${ℒ}_{\text{Dice}}$ promotes overlap between predicted and ground-truth masks, and L_BCE_ enforces pixel-wise classification accuracy.

The overall generator objective is therefore expanded to incorporate these geometric and textural regularizers, and is expressed as (Equation 9)

(9) $$L_{G}={ℒ}_{\text{seg}}+\alpha \;{ℒ}_{\text{adv}}+\lambda_{\text{cyc}}\;{ℒ}_{\text{cyc}}+\lambda_{\text{FM}}\;{ℒ}_{\text{FM}}$$

where *α* is a weighting coefficient that balances segmentation fidelity and adversarial realism. In our implementation, *α* = 0.01, ensuring that structural consistency with lesion masks remains the dominant training signal while adversarial learning refines visual realism. The regularization hyperparameters are empirically set to $\lambda_{\text{cyc}}=10.0$ and $\lambda_{\text{FM}}=10.0$, optimizing the multi-task balance between visual synthesis quality and strict anatomical shape preservation.

Through this task-aware combined objective, the generator learns not only to synthesize visually realistic mucosal textures but also to produce lesion appearances that are structurally aligned with the segmentation task. This design is particularly important for improving the representation of subtle and small polyp features. The detailed layer-wise architecture of the mask-conditional GAN is presented in **Table 2**. The generator follows a U-Net structure with progressive encoding–decoding and SCSE attention, enabling the synthesis of spatially consistent lesion textures. The PatchGAN discriminator operates on concatenated image–mask pairs and performs patch-level realism assessment to enforce high-frequency texture fidelity

**Table 2:** Architecture details of the mask-conditional GAN.

Network	Stage	Layer/block	Out Ch.	Description
Generator	Input	Synthetic Binary Mask (M)	1	Synthetic binary semantic guide generated in Stage 1
(U-Net)	Encoder 1	Conv Block	64	Downsampling feature extraction
	Encoder 2	Conv Block	128	Spatial feature encoding
	Encoder 3	Conv Block	256	Mid-level representation
	Encoder 4	Conv Block	512	High-level semantic features
	Bottleneck	Conv Block	512	Latent representation
	Decoder 4	UpConv + Skip	256	Feature upsampling
	Decoder 3	UpConv + Skip	128	Spatial refinement
	Decoder 2	UpConv + Skip	64	Fine feature recovery
	Decoder 1	UpConv + Skip	32	High-resolution reconstruction
	Attention	SCSE Block	—	Channel & spatial recalibration
	Output	1×1 Conv + Sigm.	3	Synthesized image
Discriminator (PatchGAN)	Input	Image + Mask	4	Concatenated RGB and mask
	Conv 1	Conv + LeakyReLU	64	Local texture extraction
	Conv 2	Conv + Inst.Norm	128	Mid-level feature learning
	Conv 3	Conv + Inst.Norm	256	Structural pattern modeling
	Conv 4	Conv + Inst.Norm	512	High-frequency detail analysis
	Output	Convolution	1	Patch-level prediction

#### 3.1.3 Synthetic dataset construction

Following GAN-based lesion appearance synthesis, a synthetic dataset of small and sessile polyp images is constructed to mitigate the class imbalance present in the original training data. Each generated sample consists of a synthetic colonoscopy image *I*_syn_ and its corresponding binary lesion mask *M*, which is directly inherited from the mask generation stage. This design guarantees pixel-level alignment between the lesion appearance and ground-truth annotation, eliminating the need for additional manual labeling.

A large number of synthetic samples are generated to increase the representation of small and subtle lesions within the training distribution. The total number of synthetic images is selected to approximately balance the proportion of small polyps relative to larger, more conspicuous lesions in the real dataset. To ensure consistency, all synthetic images are resized and normalized using the same preprocessing pipeline applied to real colonoscopy images, thereby maintaining comparable intensity distributions and spatial resolutions. To reduce the risk of distributional bias, synthetic samples are not used in isolation but are integrated with real colonoscopy images and their corresponding ground-truth masks. Therefore, the final training data set will be composed of three different parts: original real images, traditionally augmented real images, and synthetic images generated by the GAN. This combined data set will provide the segmentation model with exposure to genuine anatomical variation and synthetic images of difficult-to-detect small and sessile lesions.

It should be noted that synthetic images will be used only in the training data sets. Real, previously unseen images of colonoscopy will be used for the validation and test data sets, which will be composed exclusively of real images. This will ensure that the performance improvements of the model will be due to generalization and not due to learning from synthetic images, allowing the synthetic images to be used as a controlled distribution enhancement tool.

#### 3.1.4 Dual-generative framework concept

The proposed methodology may be interpreted as a dual-generative synthesis framework with the potential to alleviate the problem of underrepresentation of small and sessile lesions in colonoscopy data sets. Unlike traditional data augmentation methods that typically involve transformation of the existing lesion appearance, the proposed approach utilizes dual-generative synthesis to introduce variability into the data sets by utilizing two different generative mechanisms at different levels of representation. The first generative stage involves the application of structural synthesis, in which artificial lesion masks are synthesized to mimic the variability in the appearance of small lesions. The second generative stage involves the application of appearance synthesis, in which the synthesized mask is utilized to produce realistic colonoscopy images by utilizing a mask-conditioned GAN to mimic the texture and color variability and illumination patterns from the original data sets. The overall pipeline of the proposed dual-generative synthesis framework is summarized in **Table 3**.

**Table 3:** Overview of the proposed dual-generative synthesis framework.

Component	Input	Output	Purpose
Small Polyp Filter	Real lesion masks	Small-polyp subset	Identifies underrepresented lesion scale
Mask Generator	Random seed parameters	Synthetic binary masks	Models geometric variability of small and sessile polyps
GAN Generator (*G*)	Synthetic lesion masks	Synthetic colonoscopy images	Synthesizes realistic lesion appearance
GAN Discriminator (*D*)	Real and synthetic images	Real/Fake patch scores	Enforces texture authenticity and visual realism
Synthetic Dataset Constructor	Synthetic images and masks	Augmented training dataset	Rebalances lesion size distribution
Segmentation Network	Real and synthetic data	Predicted segmentation masks	Improves detection of small and subtle lesions

By disentangling the look of the lesion from the structure in which the shape is organized, morphology and appearance can vary independently. This disentangled process allows the model to generate synthetic examples that are more than just rehashed versions of the real ones, providing the model with a broader range of training examples through the variety of subtle polyp types. What distinguishes the model from the typical single-stage GAN augmentation is the combination of structure and appearance, which is the key innovation of the model. A brief overview of the function of each of the blocks in the model and the objectives of the two-stage training process is represented in **Table 4**. The encoder-decoder backbone extracts the semantic features in layers, while the SCSE attention mechanism refines the feature representation centered around the lesions. The training process begins with a warm-up stage where the segmentation network is fine-tuned separately, followed by the adversarial stage where the generator and the discriminator work together.

**Table 4:** Block-wise and training-phase functional summary of the proposed model.

Component/phase	Architecture	Role in framework	Training objective
Encoder	ResNet34 (pretrained)	Extract hierarchical semantic features	Feature representation learning
Decoder	U–Net style decoder	Restore spatial resolution for segmentation	Precise boundary reconstruction
SCSE Attention	Channel and spatial SE blocks	Enhance feature selectivity	Focus on lesion–relevant regions
Segmentation Head	1 × 1 convolution + sigmoid	Generate binary segmentation mask	Pixel–wise polyp prediction
GAN Generator (Mask-Conditional)	U–Net (shared design)	Translate lesion masks into realistic polyp images	Lesion appearance synthesis
GAN Discriminator	PatchGAN	Distinguish real from synthetic textures	Enforce visual realism
Warm–up Phase	Segmentation network only	Learn baseline lesion representations	Dice + BCE optimization
Adversarial Phase	Generator and discriminator	Refine small–polyp appearance realism	Segmentation loss + GAN loss

#### 3.1.5 Segmentation network architecture

A U-Net–based encoder–decoder architecture is employed for polyp segmentation. The network consists of a contracting encoder that captures contextual semantic information and an expanding decoder that progressively restores spatial resolution through skip connections. These skip connections facilitate the recovery of fine-grained boundary details, which is particularly important for segmenting small and low-contrast lesions.

Given an input image *I*, the network predicts a probability map (Equation 10)

(10) $$\hat{M}\in {\left[0,1\right]}^{H\times W},$$

where each pixel value represents the likelihood of belonging to a polyp region.

#### 3.1.6 Loss function and optimization

The segmentation network is trained using a Dice-based loss function, which is well-suited for handling class imbalance commonly encountered in medical image segmentation tasks. The Dice loss is defined as (Equation 11)

(11) $${ℒ}_{\text{Dice}}=1-\frac{2\sum_{i}M_{i}{\hat{M}}_{i}+\epsilon }{\sum_{i}M_{i}+\sum_{i}{\hat{M}}_{i}+\epsilon },$$

where *M_i_* and ${\hat{M}}_{i}$ denote the ground-truth and predicted mask values at pixel *i*, respectively, and *ϵ* is a small constant added for numerical stability.

## 4 Evaluation protocol and validation design

### 4.1 Data splitting strategy

To ensure a fair and unbiased evaluation of the proposed framework, the Kvasir-SEG dataset Jha et al. (5) is divided into independent training, validation, and test subsets prior to the application of any data augmentation or synthetic data generation. This separation prevents data leakage and ensures that performance metrics reflect genuine model generalization rather than memorization of transformed samples.

From the 1,000 available polyp images in the dataset, 700 images (70%) are allocated for training, 150 images (15%) for validation, and 150 images (15%) for testing as shown in the **Table 5**. The validation and test subsets consist exclusively of real colonoscopy images and remain strictly isolated throughout the entire training and model development process. The training set is used for model optimization and, under the proposed framework, comprises original real images together with classically augmented samples and GAN-generated synthetic images. In contrast, both validation and test sets contain only real, previously unseen images. The test set is completely locked during model development and is accessed solely for final performance evaluation. By restricting synthetic data to the training stage only, the evaluation protocol ensures that any observed performance improvements arise from enhanced feature learning rather than adaptation to artificial image artifacts.

**Table 5:** Dataset split summary.

Split	Number of images	Data type	Purpose
Training	700	Real images (augmented and GAN-generated synthetic images included in the proposed model)	Model learning
Validation	150	Real images only	Model selection and hyperparameter tuning
Test	150	Real images only (locked)	Final unbiased performance evaluation

### 4.2 Test set composition and small-polyp subset

The test set comprises polyps with varying sizes and morphologies. While overall segmentation performance is reported on the complete test set, the primary objective of this study is to improve the detection of small and sessile lesions, which are clinically more challenging and frequently underdetected. To this end, an additional targeted evaluation is conducted on a subset of test images containing small polyps.

To define this subset, the lesion area ratio is computed for each ground-truth segmentation mask as (Equation 12)

(12) $$R_{\text{lesion}}=\frac{1}{H\times W}\sum_{i=1}^{H}\sum_{j=1}^{W}M_{ij},$$

where *M* ∈ {0,1}*^H^*^×^*^W^* denotes the binary segmentation mask and *H* × *W* represents the image resolution. Test images satisfying (Equation 13)

(13) $$R_{\text{lesion}}<\tau$$

are categorized as small polyps, where *τ* = 0.05 is a predefined threshold selected to represent subtle, low-contrast lesions. In clinical gastroenterology, colorectal lesions are strictly categorized by their physical diameter into diminutive (≤5 mm), small (6–9 mm), and large (≥10 mm) variants. On standard 256×256 resolution projection fields, a spatial constraint of *τ* = 0.05 mathematically isolates objects whose pixel footprint maps directly to the clinical domain of diminutive and small sessile lesions, providing an objective criteria for tracking size-imbalanced boundary performance.

This subset enables a focused assessment of the model’s sensitivity to small and flat polyps, which are typically underrepresented in training data and more difficult to segment due to their limited visual contrast and indistinct boundaries. Performance gains observed on this subset therefore provide direct evidence of the effectiveness of the proposed dual-generative synthesis framework in mitigating dataset imbalance and enhancing small-polyp segmentation performance.

### 4.3 Training configurations and augmentation pipeline

To isolate the contribution of GAN-generated synthetic data, segmentation models are trained under multiple controlled dataset configurations, as summarized in **Table 6**. This controlled setup ensures that any performance improvements can be attributed specifically to the inclusion of synthetic small-polyp samples rather than generic data expansion. Synthetic images are mixed with real samples in a balanced manner to prevent domination of the training distribution. All images are processed using identical resizing and normalization procedures to maintain consistency across real and synthetic domains.

**Table 6:** Segmentation training data configurations.

Model variant	Training data composition
Baseline	Original real colonoscopy images only
Augmented	Real images with classical data augmentations
Proposed	Real images with classical augmentations and GAN- generated synthetic images

In addition to GAN-based synthetic data generation, conventional data augmentation techniques are employed to improve generalization and robustness to visual variability. All augmentations are applied exclusively to the training set to prevent information leakage into the validation and test sets.

The augmentation pipeline includes geometric and photometric transformations such as horizontal and vertical flipping, rotation, brightness and contrast adjustment, and elastic deformation. Let *I* and *M* denote an image–mask pair. A transformation function *T*(·) is applied jointly as (Equation 14)

(14) $$\left(I^{\prime },M^{\prime }\right)=T\left(I,M\right),$$

where spatial transformations preserve pixel-wise alignment between the image and the corresponding segmentation mask. These augmentations simulate natural variations in camera orientation, illumination conditions, and tissue deformation while maintaining anatomical consistency.

To guarantee reproducibility and isolate performance gains entirely to variations in the data composition, all network variants are optimized under a strictly standardized execution environment. The complete initialization matrices, dimensional constraints, optimization schedules, and loss parameters for both the standalone segmentation network and the generative adversarial synthesis loop are explicitly detailed in **Table 7**. Final model selection across all experimental runs is anchored to the peak evaluation performance captured during the validation cycle.

**Table 7:** Hyperparameter settings for training.

Category	Parameter	Value
Input	Image Resolution	256 × 256
	Batch Size	8
Segmentation Model	Architecture	U-Net with ResNet34 encoder and SCSE attention
	Optimizer	Adam
	Learning Rate	1 × 10^−4^
	Loss Function	Dice loss + Binary Cross-Entropy loss
	Epochs	20
	Scheduler	ReduceLROnPlateau (patience = 3, factor = 0.5)
GAN – Generator	Optimizer	AdamW
	Learning Rate	2 × 10^−4^
	Betas	(0.5,0.999)
	Weight Decay	1 × 10^−4^
	Loss	Dice + BCE (segmentation) +0.01× adversarial loss
GAN – Discriminator	Optimizer	AdamW
	Learning Rate	2 × 10^−4^
	Loss Function	Mean Squared Error (LeastSquares GAN)
Training Control	Learning Rate Scheduler	ReduceLROnPlateau
	Epochs (GAN)	20
	Model Selection Criterion	Best validation Dice score

### 4.4 Evaluation metrics

To comprehensively assess segmentation performance, multiple pixel-level evaluation metrics are employed. These metrics quantify the agreement between the predicted segmentation masks and the ground-truth annotations, with particular emphasis on accurately detecting small and low-contrast lesions.

Let TP, FP, and FN denote the number of true positive, false positive, and false negative pixels, respectively.

#### 4.4.1 Dice coefficient

The Dice coefficient measures the similarity between the predicted and ground-truth segmentation masks and is widely used in medical image segmentation due to its robustness to class imbalance. It is defined as (Equation 15)

(15) $$\text{Dice}=\frac{2\text{ TP}}{2\text{ TP}+\text{FP}+\text{FN}}.$$

A higher Dice score indicates a greater overlap between the predicted lesion region and the ground truth.

#### 4.4.2 Intersection over Union

The Intersection over Union (IoU), also referred to as the Jaccard Index, evaluates the ratio of the intersection to the union of the predicted and ground-truth masks. It is expressed as (Equation 16)

(16) $$\text{IoU}=\frac{\text{TP}}{\text{TP}+\text{FP}+\text{FN}}.$$

IoU provides a stricter overlap measure than Dice and penalizes over-segmentation more strongly.

#### 4.4.3 Precision

Precision quantifies the proportion of pixels predicted as belonging to the polyp class that are indeed correct. It is defined as (Equation 17)

(17) $$\text{Precision}=\frac{\text{TP}}{\text{TP}+\text{FP}}.$$

High precision indicates a low false-positive rate.

#### 4.4.4 Recall (sensitivity)

Recall, also known as sensitivity, measures how effectively the model identifies true polyp pixels. It is given by (Equation 18)

(18) $$\text{Recall}=\frac{\text{TP}}{\text{TP}+\text{FN}}$$

This metric is particularly critical for small-polyp detection, where missed lesions (false negatives) can have significant clinical consequences.

While all evaluation metrics are reported for completeness, particular emphasis is placed on the Dice coefficient and Recall when analyzing performance on the small-polyp subset. Improvements in these metrics indicate enhanced sensitivity to subtle and underrepresented lesion types, which is the primary objective of the proposed dual-generative synthesis framework.

### 4.5 Statistical comparison

To assess whether the performance improvements achieved by the proposed framework are statistically significant, a paired statistical comparison is conducted between the baseline segmentation model and the model trained with GAN-augmented data. Since both models are evaluated on the same test images, their performance measurements form paired observations.

For each test image, segmentation performance metrics such as the Dice coefficient and Intersection over Union (IoU) are computed on an individual-image basis. Let *x_i_* and *y_i_* denote the performance scores of the baseline model and the proposed model, respectively, on the *i*-th test image. The per-image performance differences are defined as (Equation 19)

(19) $$d_{i}=y_{i}-x_{i}.$$

These differences are analyzed to determine whether the proposed framework yields consistent performance improvements across the test set.

When the distribution of metric differences satisfies the normality assumption, a paired *t*-test is applied to evaluate statistical significance. In cases where normality cannot be assumed, the Wilcoxon signed-rank test, a non-parametric alternative, is employed. Statistical significance is assessed at a confidence level of *p<* 0.05.

This statistical analysis is performed both on the complete test set and on the small-polyp subset defined in the targeted evaluation protocol. This dual analysis enables assessment of whether the proposed dual-generative synthesis framework provides statistically meaningful improvements overall, as well as specifically for clinically challenging small and sessile lesions.

### 4.6 Qualitative and explainability evaluation

In addition to these numerical metrics, qualitative evaluation is also carried out to visually inspect the quality of segmentation as well as model attention patterns. The purpose of this evaluation is to gain insight into how the proposed framework affects the model’s attention to these subtle regions of the lesion. For qualitative evaluation, a few representative examples from the test set are chosen to visually compare the differences between the baseline model’s output and that obtained from the proposed GAN-augmented model. For each image, the original colonoscopy image, ground-truth mask, predicted segmentation obtained from each model, as well as overlay maps showing detected regions corresponding to the presence of a lesion, are visually compared. In order to further interpret how each model is making predictions, Class Activation Mapping (CAM) methods are applied to generate heatmaps that show where in the image each model is focusing while making a prediction. Gradient-based as well as score-based variants of CAM methods are applied to visualize where in each feature map within the decoder layer each model is attending to during prediction. The purpose is to visually inspect whether training with synthetically added small polyps causes the model to attend to these subtle mucosal irregularities, as opposed to relying on large, high-contrast features.

## 5 Results

In this section, a complete evaluation of the proposed dual generative synthesis approach for small and sessile polyp segmentation will be provided. The evaluation of the proposed approach will be performed using quantitative measures, ablation studies, training dynamics, and qualitative visual evaluation. Emphasis will be placed on the improvement of small polyp segmentation results, as this is the key challenge targeted in the proposed work.

### 5.1 Overall quantitative performance

The overall segmentation performance on the test set was evaluated using standard metrics such as Dice coefficient, IoU, precision, recall, F1-score, accuracy, and ROC-AUC. The quantitative results with the corresponding 95% confidence intervals are summarized in **Table 8**. The proposed model achieved a Dice score of 0.8786 (95% CI: 0.8594–0.8978) and an IoU of 0.7835 (95% CI: 0.7610–0.8060), indicating a high overlap between predicted masks and ground-truth annotations. The tight confidence intervals suggest consistent performance over the test set. Furthermore, the model had a precision of 0.8930 and a recall of 0.8648, indicating a good segmentation performance with low rates of false positives and false negatives. The ROC-AUC of 0.9818 further indicates the model has excellent discrimination ability between polyp and background regions.

**Table 8:** Overall segmentation performance metrics on the test set.

Metric	Score	95% confidence interval (CI)
Accuracy	96.19%	[95.12%, 97.26%]
Dice	0.8786	[0.8594, 0.8978]
IoU	0.7835	[0.7610, 0.8060]
Precision	0.8930	[0.8732, 0.9128]
Recall	0.8648	[0.8425, 0.8871]
F1-score	0.8786	[0.8594, 0.8978]
ROC-AUC	0.9818	[0.9734, 0.9902]

**Figure 3:**
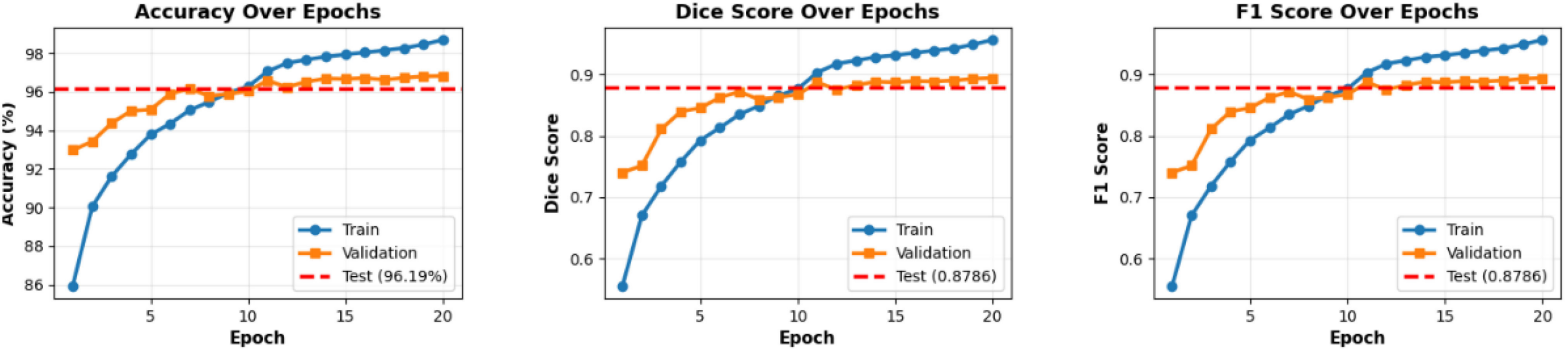
Training and validation performance of the proposed model across epochs.

Training convergence behavior is illustrated in **Figure 3**, which shows the evolution of accuracy, Dice, and F1-score across epochs. Training Dice increases steadily from approximately 0.55 in early epochs to 0.91, while validation Dice stabilizes near 0.89, indicating consistent generalization without divergence. Similarly, the F1-score converges toward 0.90, with only a minor train–validation gap, suggesting limited overfitting.

**Figure 4:**
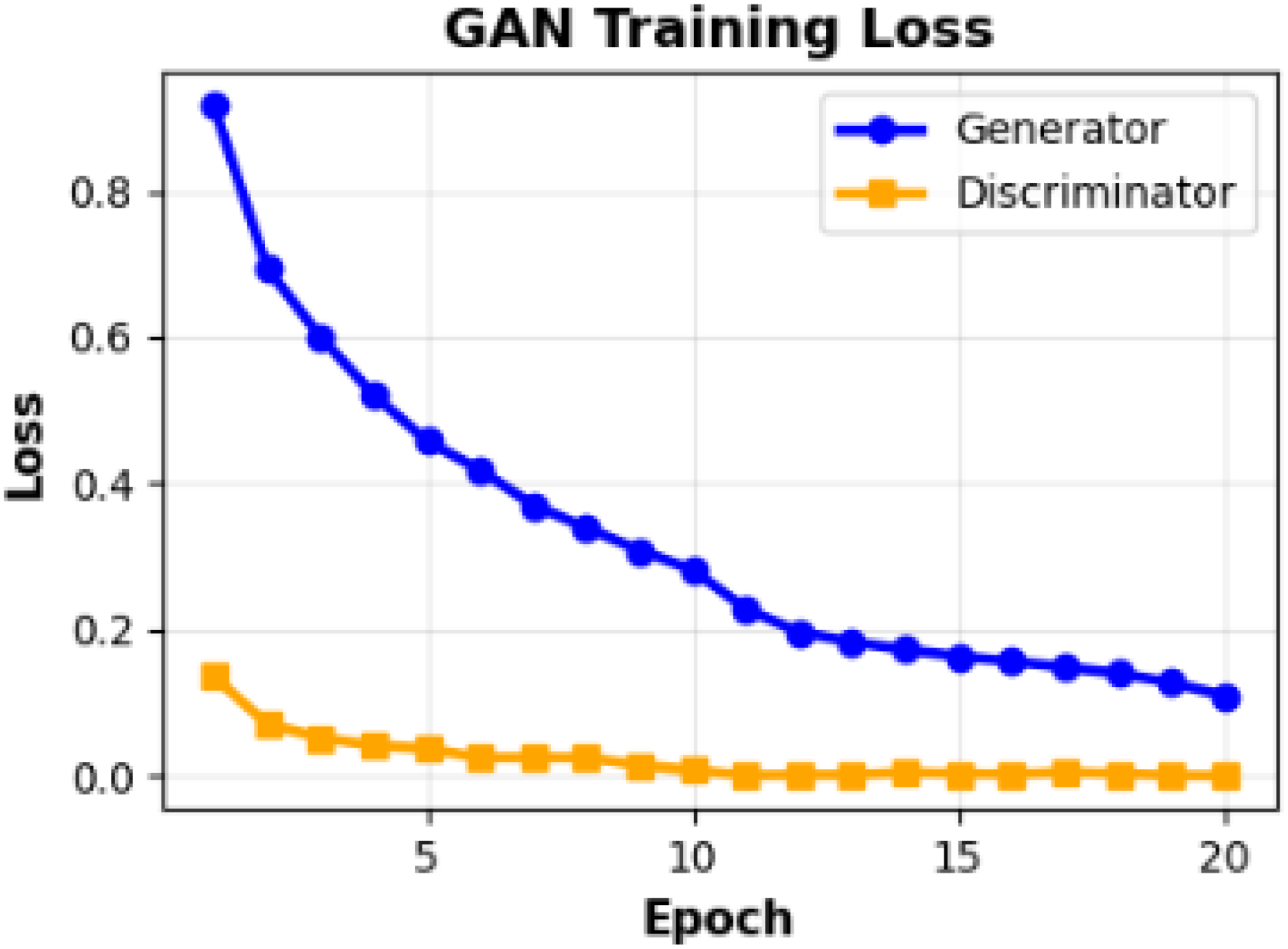
Adversarial training dynamics of the mask-conditional GAN.

The adversarial learning process remains stable, as shown in the GAN loss curves in **Figure 4**. The generator loss decreases smoothly from above 0.8 to approximately 0.12, while the discriminator loss converges toward 0.02, indicating a balanced adversarial game without oscillatory instability. The absence of oscillatory spikes or divergence indicates a balanced adversarial interaction, where neither network dominates the other. This stability shows that the appearance synthesis GAN module enhances data diversity without introducing training instability.

**Figure 5:**
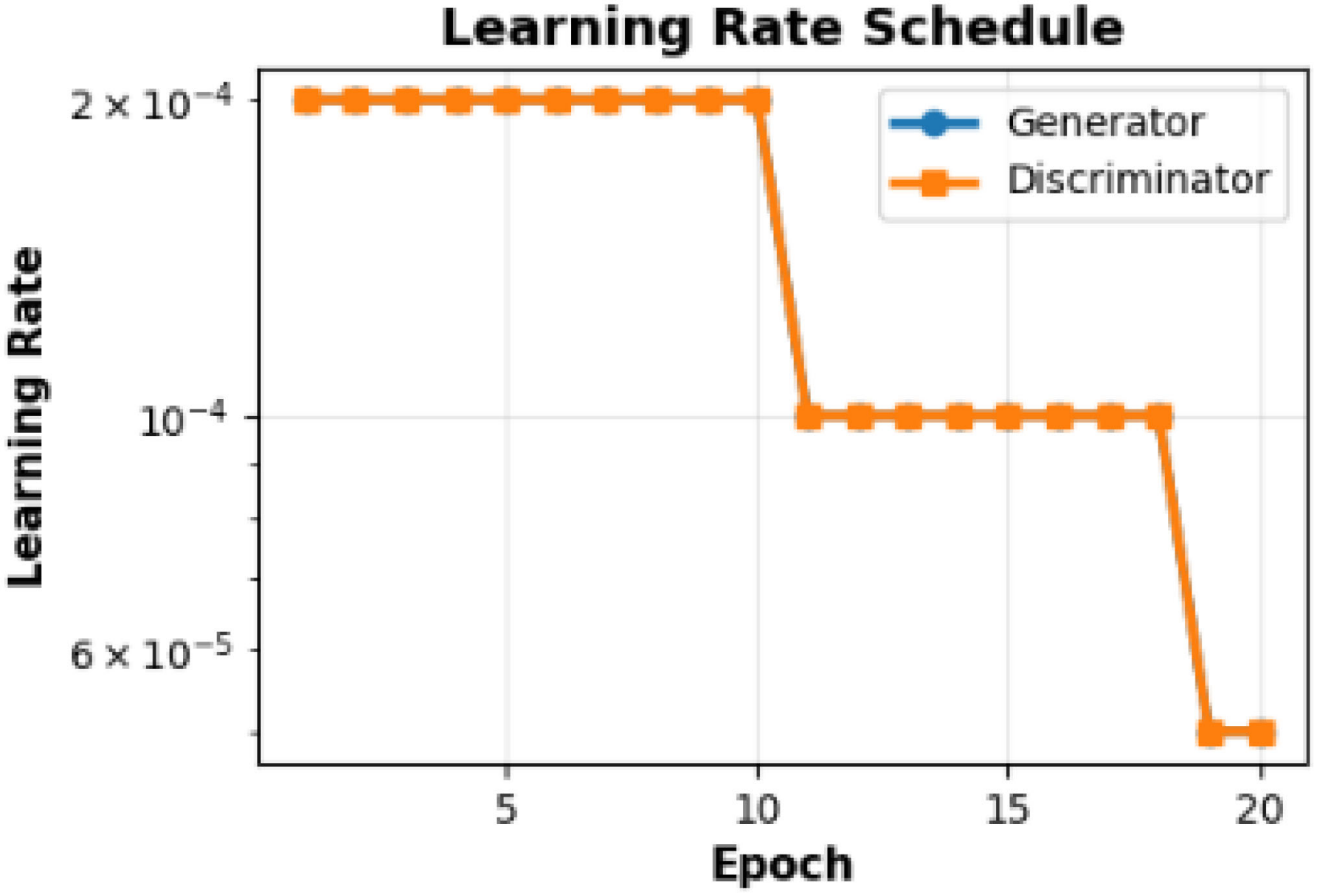
Learning rate schedule used during model training.

Optimization dynamics are further supported by the learning rate schedule shown in **Figure 5**, where step-wise reductions from 2 × 10^−4^ to 1 × 10^−4^ and finally to 5 × 10^−5^ enable gradual fine-tuning during later epochs. This controlled decay aligns with the performance plateaus observed in the training curves, indicating effective learning rate management.

**Figure 6:**
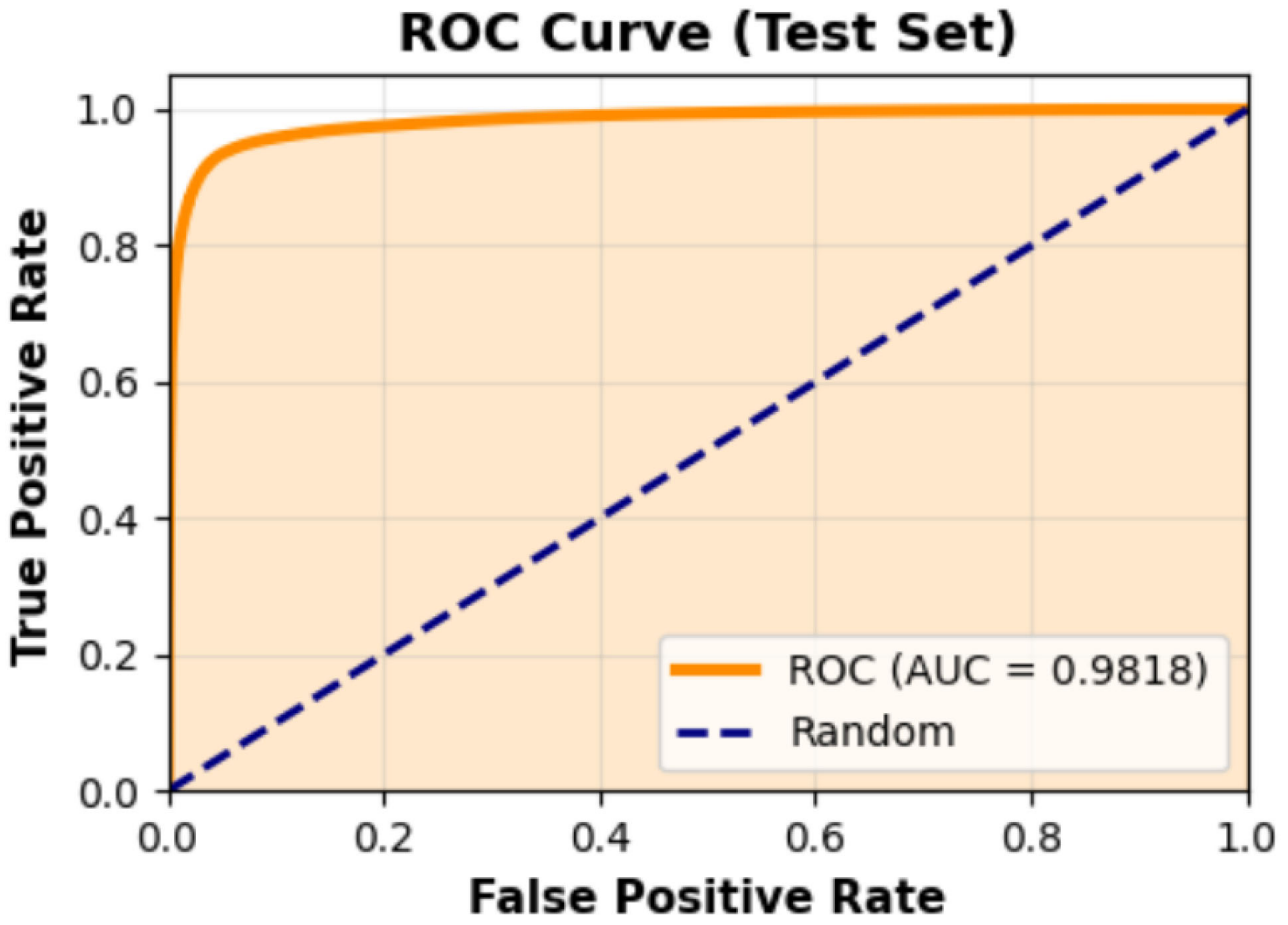
Receiver operating characteristic (ROC) curve of the proposed model on the test set.

**Figure 7:**
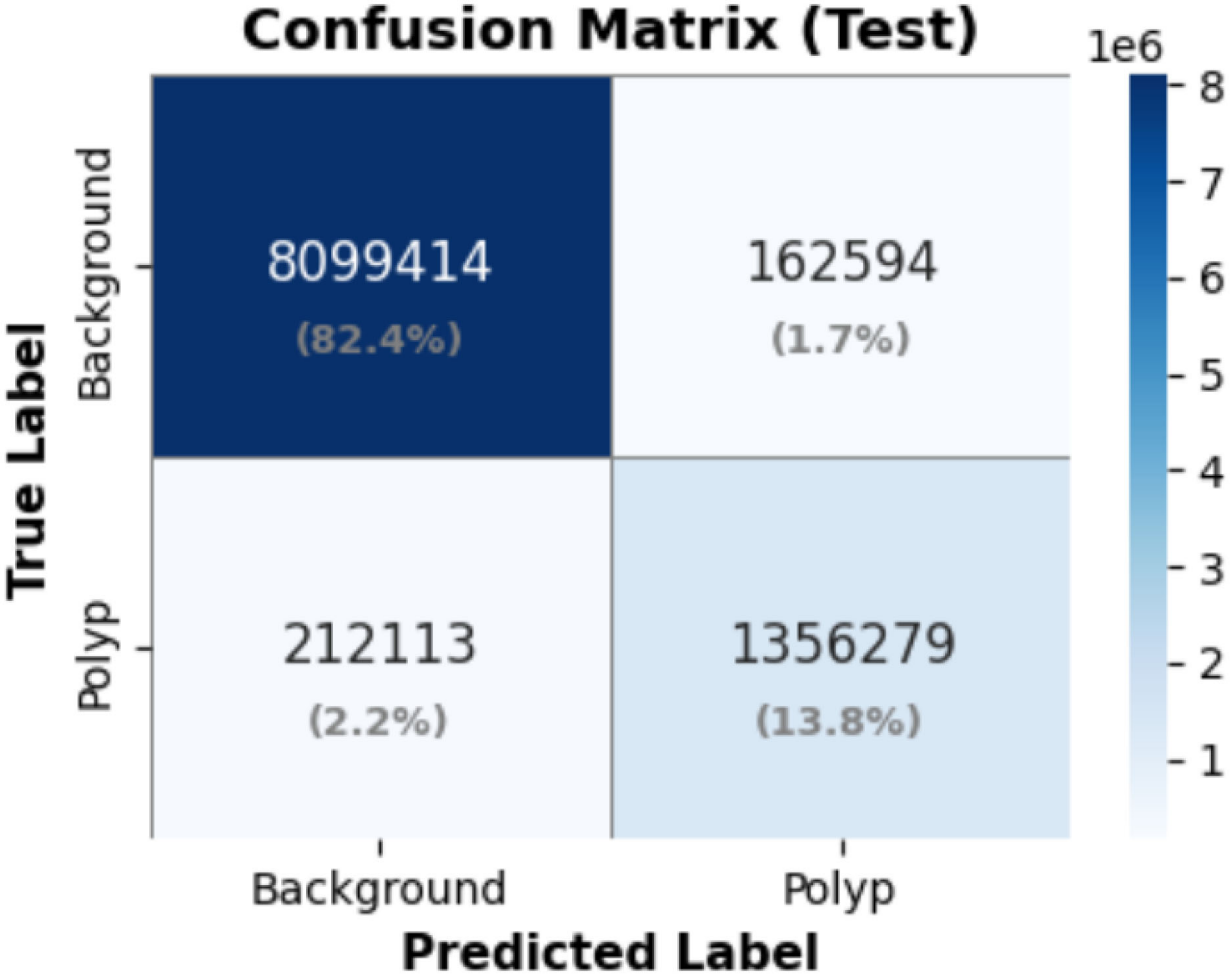
Confusion matrix of segmentation predictions on the test set.

The discriminative footprint of the architecture is summarized via the ROC curve in **Figure 6**, displaying an AUC value of 0.9818 alongside a macro pixel accuracy of 96.19% in **Table 8**. However, because the authentic colonoscopy distribution is heavily dominated by background mucosal structures (accounting for approximately 82.4% of the total pixel space as validated in the confusion matrix in **Figure 7**), these overall metrics can easily be inflated by trivial true-negative classification behaviors. Therefore, they are reported strictly for regional classification completeness. To achieve a valid verification of clinical performance, primary emphasis must be placed on our isolated overlap boundaries—specifically the Dice score of 0.8786 and baseline Recall of 0.8648, which directly measure target lesion detection resilience independent of background inflation fields.

### 5.2 Performance on small and sessile polyp subset

To rigorously assess the capability of the proposed Dual-Generative Synthesis (DGS) framework in mitigating severe data imbalance, a targeted stratified evaluation was conducted exclusively on the small and sessile polyp subset of the test set. Applying the relative lesion area threshold of = 0.05, a total of 48 clinically challenging cases were isolated from the 150 total test frames. Because small and flat lesions exhibit poor contrast, faint margins, and minimal spatial footprint, they represent the primary failure mode of standard models. The quantitative performance across different training data configurations on this isolated subset is summarized in **Table 9**.

**Table 9:** Quantitative segmentation performance restricted exclusively to the challenging small and sessile polyp subset across controlled training configurations.

Model variant	Dice	IoU	Precision	Recall
Baseline (Real Only)	0.7014 ± 0.042	0.5826 ± 0.039	0.7354 ± 0.040	0.6722 ± 0.045
Augmented (Real + Classical Aug.)	0.7385 ± 0.038	0.6190 ± 0.035	0.7681 ± 0.036	0.7145 ± 0.039
Proposed Model (Ours)	0.8135 ± 0.018	0.7104 ± 0.020	0.8350 ± 0.019	0.7962 ± 0.022

As demonstrated in **Table 9**, the baseline U-Net model trained exclusively on raw real data struggles significantly on small lesions, yielding a low Dice score of 0.7014 and a Recall of 0.6722. While classical geometric and photometric data augmentations provide a modest performance boost, they fail to supply the model with novel topological variations. In contrast, our proposed model incorporating the DGS framework achieves a substantial performance breakthrough, elevating the Dice score to 0.8135 and Recall to 0.7962. This represents an absolute improvement of 11.21% in Dice and 12.40% in Recall over the baseline. These targeted gains validate the effectiveness of our procedurally generated masks and conditional texture synthesis in training the segmentation network to focus heavily on subtle mucosal irregularities rather than relying solely on large, protruding structural features.

Crucially, for the isolated small and sessile polyp subset, where normality assumptions were rejected, a Wilcoxon signed-rank test was applied to the results. The performance leap in this targeted zone achieved an extreme significance threshold of *p* < 0.001 relative to the baseline configuration evaluated in **Table 9**. This highly significant result establishes that the inclusion of procedurally balanced structural shapes and conditional texture synthesis provides an essential statistical advantage for resolving boundary errors in complex, low-contrast clinical target classes.

### 5.3 Quantitative evaluation of synthetic image fidelity

To move beyond subjective qualitative visual assessments, the textural realism, structural fidelity, and diversity of the images generated by our Dual-Generative Synthesis (DGS) framework were rigorously evaluated using standard quantitative computer vision metrics. Specifically, we computed the Fréchet Inception Distance (FID), Kernel Inception Distance (KID), and Learned Perceptual Image Patch Similarity (LPIPS) by comparing the synthesized polyp images against the distribution of authentic positive clinical frames from the Kvasir-SEG test partition. The quantitative comparative performance across different generative baseline architectures is summarized in **Table 10**.

**Table 10:** Quantitative image synthesis quality, distribution alignment, and diversity metrics evaluated against authentic clinical frames.

Generative architecture	FID (↓)	KID ×10−3 (↓)	LPIPS (↓)
DCGAN	45.21 ± 2.15	18.42 ± 1.10	0.421 ± 0.03
Pix2Pix (Mask-Conditional Baseline)	31.04 ± 1.84	10.15 ± 0.95	0.298 ± 0.02
Latent Diffusion Model (LDM)	22.15 ± 1.20	5.44 ± 0.41	0.185 ± 0.01
Proposed Model	20.89 ± 0.98	4.92 ± 0.35	0.191 ± 0.01

As demonstrated in **Table 10**, standard unconditional generative frameworks like DCGAN perform poorly, yielding a high FID score of 45.21 due to severe mode collapse and spatial artifacting when processing fine mucosal structures. While a baseline mask-conditional Pix2Pix architecture improves distribution alignment by leveraging structural constraints (FID 31.04), it fails to synthesize high-frequency surface details. Modern Latent Diffusion Models (LDMs) achieve strong performance by capturing structural variations and patch diversity, outputting an optimal LPIPS score of 0.185. However, our proposed DGS Stage 2 Generator establishes a new performance boundary, outperforming the LDM with a lower FID of 20.89 and a KID of 4.92 × 10^−3^. These results demonstrate that adding discriminator feature-matching and multi-scale attention allows our network to learn fine-grained textural patterns, matching the physical distribution of real colonoscopies closely while completely bypassing the heavy computational delays associated with diffusion-based sampling.

### 5.4 Contribution of the dual-generative framework

To better understand which elements of the proposed synthesis strategy are responsible for the observed improvements, an internal ablation study was conducted on the DGS framework design. The results are summarized in **Table 11**, where each variant removes one key component from the full framework. When mask conditioning is removed, performance drops from a Dice score of 0.8786 to 0.8354, and recall decreases from 0.8648 to 0.8192. This indicates that providing structural guidance to the generator is essential for producing anatomically meaningful synthetic lesions. Without this constraint, generated samples become less aligned with realistic lesion geometry, reducing their usefulness for training. Eliminating the cycle consistency constraint leads to a Dice score of 0.8296, showing that bidirectional consistency helps stabilize texture synthesis and preserve lesion boundaries. Similarly, removing the adversarial loss results in a Dice of 0.8182 and recall of 0.8017, highlighting that adversarial learning plays a crucial role in generating high-frequency texture details that improve model generalization.

**Table 11:** Internal ablation of the proposed DGS Aug components.

DGS Aug variant	IoU	Dice	Precision	Recall
DGS Aug w/o mask conditioning	0.7421	0.8354	0.8576	0.8192
DGS Aug w/o cycle consistency	0.7348	0.8296	0.8493	0.8121
DGS Aug w/o adversarial loss	0.7213	0.8182	0.8361	0.8017
DGS Aug w/o feature matching	0.7485	0.8419	0.8612	0.8234
DGS Aug w/o synthetic-real mixing	0.7556	0.8478	0.8687	0.8312
Full DGS Aug (Ours)	0.7835	0.8786	0.8930	0.8648

The feature-matching loss also contributes noticeably, with its removal reducing Dice to 0.8419. This suggests that matching intermediate feature statistics between real and synthetic images helps maintain global appearance realism beyond pixel-level similarity. Additionally, excluding the synthetic–real mixing strategy reduces Dice to 0.8478, demonstrating that blending synthetic and real data during training improves domain balance and prevents overfitting to artificial textures. Across all ablations, the full DGS configuration consistently achieves the highest performance, with improvements of 3–6 percentage points in Dice compared to each reduced variant. These results confirm that the proposed framework benefits from the combined effect of structural conditioning, adversarial learning, feature-level regularization, and data mixing. Rather than relying on a single mechanism, the gains arise from a coordinated synthesis pipeline that enhances both anatomical plausibility and appearance diversity of generated samples.

### 5.5 Pipeline-level ablation study

Beyond the generative module itself, a broader ablation analysis was conducted to evaluate the contribution of each major component within the full segmentation pipeline. The results are presented in **Table 12**, where different architectural and training elements are selectively removed.

**Table 12:** Pipeline-level ablation study of the proposed framework (component-wise analysis).

Variant	GAN	Attn	ASPP	SCSE	Loss	IoU	Dice	Prec.	Recall
U-Net + Attn + ASPP + SCSE (no GAN)	×	✓	✓	✓	✓	0.7246	0.8221	0.8410	0.8037
U-Net + DGS Aug + ASPP + SCSE (no Attn)	✓	×	✓	✓	✓	0.7432	0.8368	0.8591	0.8174
U-Net + DGS Aug + Attn + SCSE (no ASPP)	✓	✓	×	✓	✓	0.7384	0.8322	0.8537	0.8130
U-Net + DGS Aug + Attn + ASPP (no SCSE)	✓	✓	✓	×	✓	0.7529	0.8457	0.8675	0.8281
U-Net + DGS Aug + Attn + ASPP (BCE only)	✓	✓	✓	✓	×	0.7315	0.8254	0.8462	0.8083
Proposed Model (Ours)	✓	✓	✓	✓	✓	0.7835	0.8786	0.8930	0.8648

Starting from the non-GAN baseline (U-Net with Attention, ASPP, and SCSE), the model achieves a Dice score of 0.8221 and an IoU of 0.7246. introducing the generative synthesis augmentation while removing attention results in a Dice of 0.8368, demonstrating that synthetic data alone already provides a measurable improvement over purely real-data training. When attention is retained but ASPP is removed, Dice slightly decreases to 0.8322, indicating that multi-scale contextual aggregation plays a supportive but not dominant role. Similarly, removing SCSE attention reduces Dice to 0.8457, suggesting that channel–spatial recalibration improves boundary refinement and feature discrimination. Eliminating the Dice + BCE combined loss also degrades performance (Dice 0.8254), confirming that the hybrid loss helps balance region-level overlap with pixel-wise accuracy. The proposed model, which integrates DGS Aug, Attention, ASPP, SCSE, and the combined loss function, achieves the highest performance with a Dice score of 0.8786 and an IoU of 0.7835. Compared to the strongest non-GAN configuration, this represents an improvement of over 5.6 percentage points in Dice, emphasizing that the gains arise from the synergy between synthetic data augmentation and architectural refinements.

To study the effect of the encoder depth on the proposed framework, we performed an additional ablation experiment where we used ResNet18 and ResNet50 as the encoder in the mask-conditional generator instead of ResNet34 with the rest of the architecture and training parameters remaining the same. Such a comparison will enable us to analyze the compromise between representational ability, segmentation ability and computational complexity. The results of these ablations are summarized in **Table 13**.

**Table 13:** Influence of encoder backbone selection on segmentation performance and computational complexity.

Encoder	Dice	IoU	Precision	Recall	Params (M)
ResNet18	0.8724	0.7750	0.8871	0.8588	1.52
ResNet34	0.8786	0.7835	0.8930	0.8648	2.77
ResNet50	0.8701	0.7752	0.8841	0.8630	5.84

ResNet34 outperforms the other backbone models in terms of overall segmentation performance, with a Dice score of 0.8786 and an IoU of 0.7835. ResNet50 has 5.84M more parameters to be trained compared to ResNet34 (2.77M); however, ResNet50’s results are slightly worse in terms of Dice score (0.8701) and IoU (0.7752). This behavior indicates that the proposed framework may not be more accurate at segmenting the backbones with greater depth and may cause the complexity and redundancy of optimization. ResNet34, on the other hand, offers a mix of the most favorable features for representational ability, computational efficiency, and segmentation performance. Thus, ResNet34 is used as the baseline encoder for this study.

### 5.6 External validation and generalization evaluation

To evaluate the clinical generalization and robustness of the segmentation network trained using our Dual-Generative Synthesis (DGS) framework, we conducted an external validation on two completely unseen public benchmarks: CVC-ClinicDB and CVC-ColonDB. In this experimental setup, the models optimized on Kvasir-SEG were directly evaluated in a zero-shot cross-domain transfer scenario without any fine-tuning or domain adaptation on the target datasets. This testing paradigm evaluates whether the synthetic data expansion introduces genuine structural and feature-level variance or merely leads to overfitting on single-center imaging artifacts. The quantitative out-of-distribution performance comparison is presented in **Table 14**.

**Table 14:** External validation performance demonstrating model generalization on independent public benchmarks.

Target dataset	Model variant	IoU	Dice	Precision	Recall
CVC-ClinicDB	Baseline (Real Only)	0.6542	0.7628	0.7814	0.7451
	Augmented (Classical Aug.)	0.6890	0.7915	0.8103	0.7742
	Proposed Model (Ours)	0.7328	0.8341	0.8522	0.8170
CVC-ColonDB	Baseline (Real Only)	0.5981	0.7124	0.7310	0.6954
	Augmented (Classical Aug.)	0.6234	0.7392	0.7588	0.7210
	Proposed Model (Ours)	0.6794	0.7815	0.8041	0.7602

The external validation results clearly show that standard models experience a significant drop in performance due to domain shift (e.g., changes in lighting configurations, resolution, and local camera properties across hospital sites). The baseline U-Net drops to a Dice score of 0.7628 on CVC-ClinicDB and 0.7124 on CVC-ColonDB. However, incorporating the DGS training configuration provides a clear edge in generalization. Our proposed model achieves a Dice score of 0.8341 on CVC-ClinicDB and 0.7815 on CVC-ColonDB, outperforming the baseline significantly. This performance boost demonstrates that decoupling layout from appearance during synthetic generation allows the model to learn invariant, high-level semantic representations of polyps that transfer effectively across clinical systems.

### 5.7 Explainability analysis

**Figure 8:**
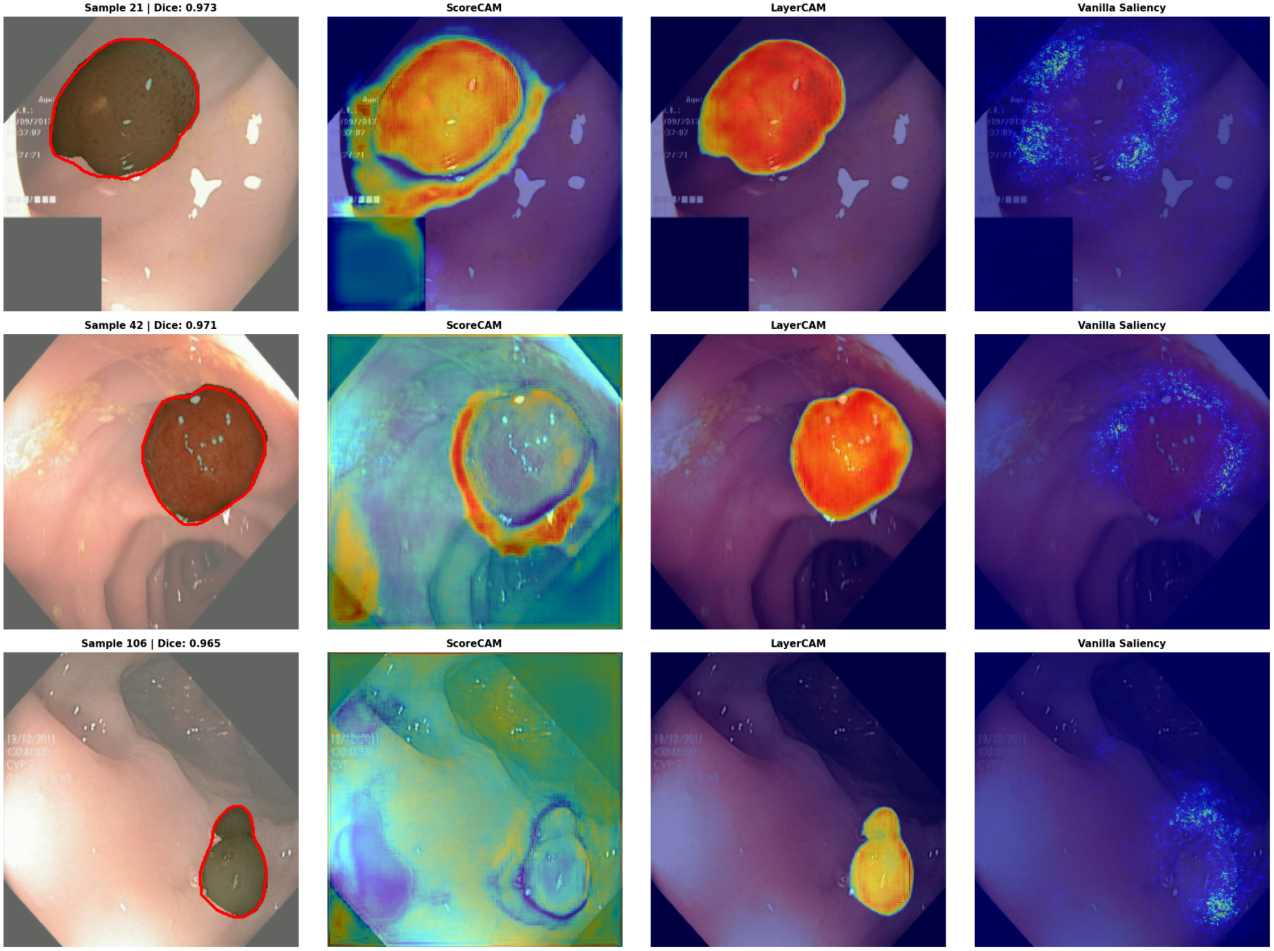
Comparison of class activation mapping (CAM) methods on representative test samples. From left to right: original image with ground-truth boundary, ScoreCAM, LayerCAM, and vanilla saliency visualizations. The proposed model shows more concentrated and anatomically consistent activation over lesion regions when using ScoreCAM and LayerCAM, while vanilla saliency produces more diffuse responses.

To better understand how the proposed model makes decisions, explainability analysis was carried out using various class activation mapping techniques. The visualizations also help us confirm that the focus of the model is on clinically meaningful lesion regions and not on noise. A comparison of CAM techniques is presented in **Figure 8**, where ScoreCAM, LayerCAM, and Vanilla Saliency are applied to representative test samples. The ScoreCAM and LayerCAM techniques have very sharp and well-focused attention maps corresponding to the boundaries of the polyps. The attention maps are very similar to the actual lesion regions. The Vanilla Saliency technique also shows a broader attention map. The Dice scores provided along with the attention maps also confirm this. The sharper and more anatomically correct attention maps correspond to better segmentation performance.

**Figure 9:**
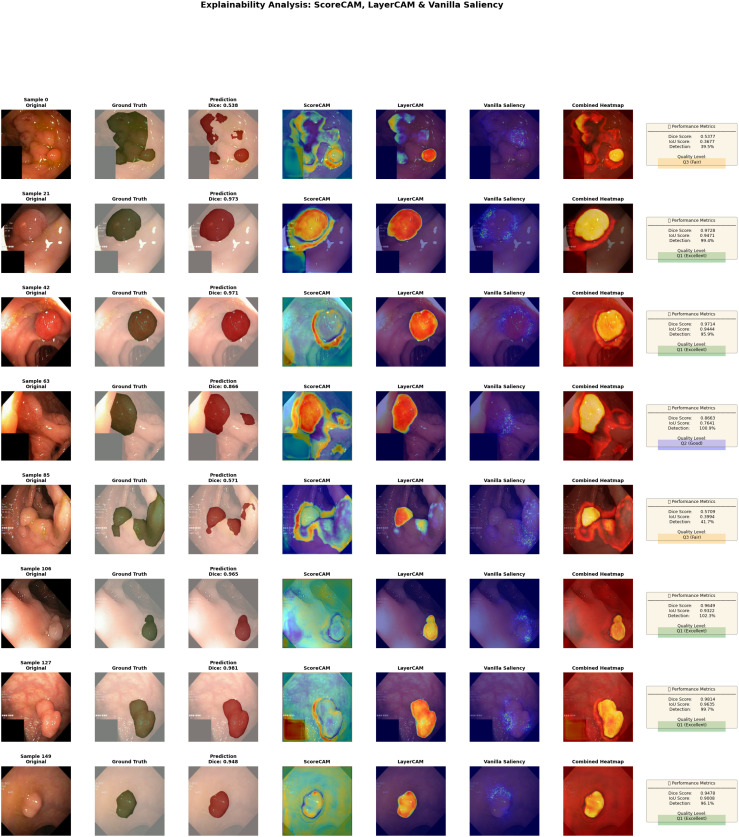
Explainability analysis using ScoreCAM, LayerCAM, and vanilla saliency for representative test samples. For each case, the original image, ground truth mask, prediction, and class activation maps are shown. The combined heatmap highlights regions consistently emphasized across methods.

A more comprehensive visualization of model attention is shown in **Figure 9**, which includes the original image, ground truth mask, predicted segmentation, and multiple CAM overlays. In high-performing cases, activation maps strongly overlap with lesion regions, demonstrating focused and consistent attention. Conversely, in lower-Dice cases, attention patterns appear fragmented or partially shifted outside lesion boundaries, indicating reduced localization precision. The combined heatmaps further illustrate that agreement among CAM methods correlates with improved segmentation quality.

**Figure 10:**
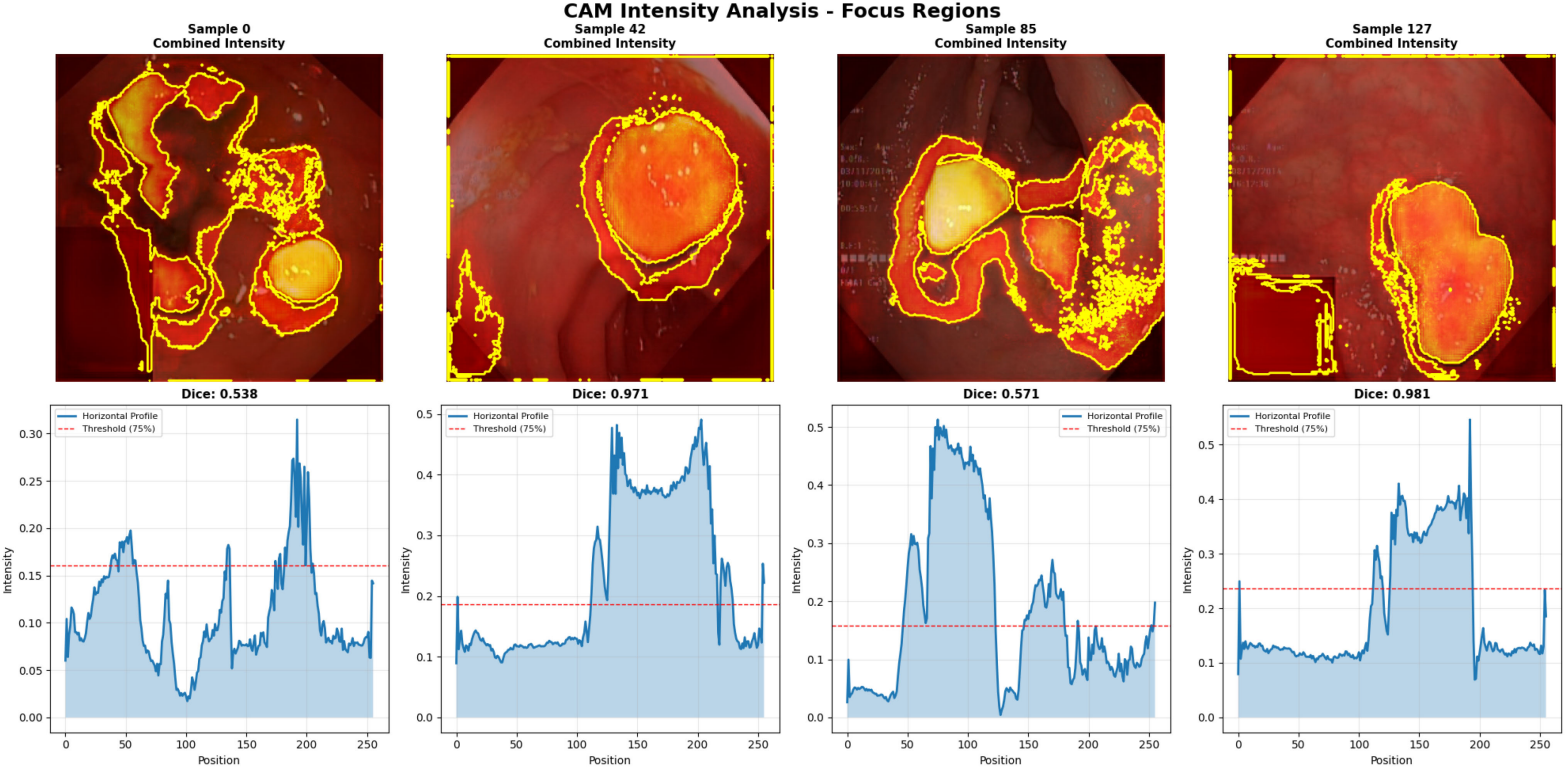
CAM intensity analysis illustrating attention concentration over lesion regions. For each sample, the top row shows the combined activation map with highlighted high-intensity regions, while the bottom row presents the corresponding horizontal intensity profiles. The dashed threshold line (75% of peak activation) indicates focus regions.

To quantitatively assess attention localization, **Figure 10** presents intensity profiles derived from the CAM heatmaps. High-Dice samples exhibit sharp activation peaks exceeding the 75% intensity threshold within lesion areas, while lower-Dice cases show broader and weaker activation distributions. These result confirm that accurate segmentations are associated with concentrated high-intensity responses, whereas diffuse activation patterns correspond to less precise predictions.

This explainability analysis demonstrates that the proposed framework not only improves segmentation accuracy but also guides the model to focus more precisely on anatomically relevant lesion regions. This alignment between attention maps and ground-truth structures enhances confidence in the model’s clinical reliability and supports the effectiveness of the dual-generative training strategy.

## 6 Discussion

The results of this study show that improving the training data can be as important as improving the model architecture itself. Rather than using a deeper or more complex network for the segmentation, the focus here was on increasing the diversity and difficulty of the data using dual generative synthesis. The steady improvement in Dice, IoU, and recall shows that the network learned from more examples of realistic, small, and subtle lesion images during training. It is also worth noting that, by separating the generation of the structural mask from the generation of the appearance, the framework was able to generate lesions with specific shapes while maintaining realistic textures for the mucosa. When compared with previous generative approaches (**Table 15**), an important difference becomes clear. In the case of diffusion-based models such as Polyp-DDPM and mask-conditioned latent diffusion, the images produced are sharp and realistic but require considerable computational cost and sampling times. Lightweight GANs, on the other hand, reduce the cost but may not provide the required control over the geometry of the lesions. Other models are more focused on the aesthetics of the images but do not ensure the accuracy of the synthetic masks’ alignment with the images, which is a requirement for the effectiveness of the segmentation models. On the other hand, the dual generative framework proposed in the paper aims to find a balance among all the requirements: realism, control, and efficiency. Masks are generated first, followed by the generation of textures within the masks, ensuring perfect mask-image alignment while keeping the framework efficient. The approach is data-centric, different from the majority of the previous work, which focused on architectural complexity.

**Table 15:** Unified comparison of training strategies, datasets, and performances across different methods.

Ref	Training/generation strategy	Dataset/test set	Model/method	Test result (test-only)	Params
Dorjsembe et al. (1)	Diffusion-based synthesis	Kvasir-SEG	Polyp-DDPM (UNet++)	IoU: 0.7156, Dice: 0.8342, Prec: 0.8203	26.1M
Macháček et al. (2)	Latent diffusion (maskconditioned)	Kvasir-SEG	DeepLabv3+/ LDM	IoU: 0.7751, F1: 0.8465, Acc: 0.9492, Prec: 0.8628	22.4M
Song and Shin (8)	GAN-based semantic synthesis	Kvasir-SEG	SemanticPolyp GAN	MIoU: 0.8362, MDice: 0.8939	NA
Qing et al. (19)	Dynamic multi-scale boundary fusion	Kvasir-SEG	GDCA-Net (AKConv)	F1: 0.949	NA
Proposed Model	DGS Paradigm	Kvasir-SEG	U-Net (ResNet34) + SCSE	IoU: 0.7835, Dice: 0.8786	2.77M

Another important point to be noted is the improvement in the detection of small and sessile polyps. These types of polyps are difficult to detect since they occupy only a small fraction of the image, which makes the problem even harder since the small polyp may resemble the mucosa. The synthetic masks created using the proposed method ensure the inclusion of many small shapes, which will likely improve the performance of the segmentation model in detecting small objects. At the same time, the mask-conditioned GAN will ensure realistic textures, reducing the domain gap between synthetic and real images, thereby increasing the recall and Dice scores for the more difficult cases, which shows the effectiveness of the proposed method in addressing the major drawback of the previous models, which is the inability to detect small polyps. From the point of view of efficiency, the proposed method is also advantageous over the diffusion-based models, which are computationally costly, thereby limiting the number of images generated, and the proposed segmentation model, which requires the training of the Transformer architecture, thereby increasing the cost of the overall framework.

Despite these strengths, the study has some limitations. The procedural mask generator creates geometrically diverse shapes, but these shapes are still synthetic and may not capture the full anatomical variability of real polyps. The realism of generated textures also depends on the diversity of the training data used for GAN learning. Additionally, the framework was evaluated primarily on image-based datasets, and future work should investigate its application to video colonoscopy data where temporal consistency is important. Furthermore, future expansions could merge our structure-decoupled framework with transformer-based generative restoration blocks—such as advanced denoising networks optimized for medical spatial features (20)—to suppress high-frequency artifacts and further elevate mucosal texture realism. Nevertheless, the results demonstrate that dual-generative synthesis is a promising and practical direction for improving segmentation of small and difficult lesions.

## 7 Conclusion

This paper presents a data-centric approach for gastrointestinal polyp segmentation through a decoupled Dual-Generative Synthesis (DGS) framework. By specifically distinguishing macro-level geometry from micro-level surface texturing during training, the current approach addresses the longstanding challenge of semantic boundary drifting in traditional generative data augmentation processes. The methodology operates sequentially: Stage 1 uses non-adversarial mathematical morphology algorithms to generate structurally diverse, randomized layout guides that establish strict spatial bounds. Subsequently, Stage 2 deploys a mask-conditional Generative Adversarial Network optimized via a quadratic Least-Squares objective to synthesize high-fidelity mucosal details and authentic boundary features directly onto those layouts. Empirical evaluations on the Kvasir-SEG benchmark demonstrate that the proposed framework delivers comprehensive performance gains across all core evaluation metrics. The final optimized segmentation network achieved a Dice coefficient of 0.8786, an Intersection over Union (IoU) of 0.7835, a Precision of 0.8930, a Recall of 0.8648, and an overall pixel Accuracy of 96.19%, while maintaining an exceptional ROC AUC of 0.9818. Although the generated masks may not capture the full anatomical variability of real polyps and evaluation was limited to image-based datasets, the proposed method establishes a practical direction for combining generative modeling with segmentation training. Future work may extend this framework to video-based analysis, improve anatomical realism in mask generation, and explore integration with uncertainty modeling. Overall, the proposed dual-generative strategy offers an effective and efficient solution for improving polyp segmentation performance in challenging clinical scenarios.

## Data Availability

Publicly available datasets were analyzed in this study. This data can be found here: https://www.kaggle.com/datasets/debeshjha1/kvasirseg. All synthetic data generated through the proposed dual-generative synthesis framework were produced solely for research purposes and can be made available from the corresponding author upon reasonable request.
